# Characterization of Azorean Plant Leaves for Sustainable Valorization and Future Advanced Applications in the Food, Cosmetic, and Pharmaceutical Industries

**DOI:** 10.3390/antiox13030325

**Published:** 2024-03-06

**Authors:** Jorge Gomes Lopes Barros, Raquel Fernandes, Ana Abraão, Rui Dias Costa, Alfredo Aires, Irene Gouvinhas, Daniel Granato, Ana Novo Barros

**Affiliations:** 1 Centre for Research and Technology of Agro-Environmental and Biological Sciences, CITAB, Inov4Agro, University of Trás-os-Montes and Alto Douro, UTAD, Quinta de Prados, 5000-801 Vila Real, Portugal; rgfernandes@utad.pt (R.F.); aabraao@utad.pt (A.A.); ruiacosta@utad.pt (R.D.C.); alfredoa@utad.pt (A.A.); igouvinhas@utad.pt (I.G.); 2 Bioactivity & Applications Laboratory, Department of Biological Sciences, Faculty of Science and Engineering, School of Natural Sciences, University of Limerick, V94 T9PX Limerick, Ireland; daniel.granato@ul.ie

**Keywords:** Azorean leaves, bioactive compounds, dietary antioxidants, sustainability, circular economy, industrial applications

## Abstract

The historical use of plants as sources of natural compounds has persisted over time. Increasing the intake of bioactive substances shows significant potential for promoting overall well-being and health. This study delves into the pigments, phenolic composition, and profile, along with antioxidant properties, of leaf extracts rich in bioactives from plants in the Azores region, contributing to sustainable primary food production. Analyses encompassed chlorophylls, carotenoids, total phenols, *ortho*-diphenols, and flavonoids, as well as antioxidant capacity assessment, polyphenolic profiling, and quantification. *Psidium guajava* L. and *Smallanthus sonchifolius* (Poepp.) H.Rob. exhibited elevated chlorophyll content, while *Colocasia esculenta* (L.) Schott displayed the highest carotenoid levels. *Annona cherimola* Mill., *Eriobotrya japonica* (Thunb.) Lindl, and *Psidium guajava* L. demonstrated pronounced total phenols, *ortho*-diphenols, and flavonoids. These findings align with heightened antioxidant capacity. HPLC–DAD (high-performance liquid chromatography with diode-array detection) characterization unveiled elevated hydroxycinnamic acids in *E. japonica* and *Ipomea batatas* (L.) Lam. compared to *A. cherimola* Mill., while *C. esculenta* exhibited increased flavone content. Among the quantified compounds, flavonols were the ones that predominantly demonstrated contribution to the antioxidant capacity of these leaves. This research highlights Azorean leaf plants’ antioxidant potential, fostering natural product development for better health.

## 1. Introduction

Plants have been globally used as abundant sources of biomolecules with potential biological functions since ancient times. Consumption of edible vegetables, especially leaves from species integrated into the human diet, is generally considered safe [1]. Despite the potential health benefits associated with bioactive substances such as chlorophylls, non-provitamin carotenoids, and phenolic compounds, precise dietary recommendations for these compounds remain elusive. However, adhering to international health organizations’ guidelines, which recommend including 400–500 g of fruits in the daily diet as part of the ‘five-a-day’ regimen, yields an estimated average daily intake of phenolic compounds ranging from 800 to 1000 mg [2]. The recommended daily intake of carotenoids is not specified as a standalone value because carotenoids are often assessed in the context of vitamin A activity, as certain carotenoids serve as precursors to vitamin A. The Recommended Dietary Allowance (RDA) for vitamin A is approximately 900 μg per day for men and 700 μg per day for women [3]. Moreover, the daily recommended intake of chlorophyll is around 50 mg chlorophylls per day [4]. 

Phenolic compounds are secondary metabolites predominantly found in plants and are produced, among other causes, in response to specific edaphoclimatic conditions [5]. Usually associated with defense responses in plants, they also play a role in mediating the incorporation of substances to accelerate pollination, contribute to coloring for camouflage, and provide protection against bacterial and fungal activities [6,7]. Phenolic compounds feature one or more hydroxyl groups attached to the *ortho*, *meta*, or *para* positions on a benzene ring. These hydroxyl groups exhibit high reactivity, readily donating electrons or hydrogens to neutralize free radicals. Aromatic groups facilitate electron delocalization, enhancing their stability. These characteristics confer potent antioxidant and anti-inflammatory properties upon these compounds [8]. Consequently, they have garnered significant research attention in various diseases and health contexts. The effect of phenolic compounds in different tumors is demonstrated by reduction in cancer cell growth, proliferation, and metastasis [9,10,11]. Additionally, these compounds display antimicrobial activity [12], possess capacity to modulate oxidative stress, lipid peroxidation [13], and prevent cardiovascular disease [14]. The incorporation of polyphenols in cosmetic industry products has also gained special interest, owing to their ability to enhance skin elasticity, alleviate aging signs, and promote collagen synthesis [15].

Chlorophylls and carotenoids are natural pigments biosynthesized in plant chloroplasts. Their function and properties are determined by their chemical structure, but their antioxidant capacity has led to an increasing presence in the daily diet [16]. Chlorophylls, cyclic tetrapyrroles crucial for photosynthesis, absorb light energy and convert it into chemical energy. Chlorophyll a and chlorophyll b have distinct structures and absorb light at different wavelengths, playing roles in light absorption and protection against excess light [17]. In vitro and in vivo studies suggest that chlorophylls exhibit antioxidant, anti-cancer, and anti-inflammatory properties, among other health benefits [18,19,20,21,22]. 

Carotenoids, which are distinguished by a polyene chain with nine conjugated double bonds, are broadly classified as carotenes and xanthophylls. The benefits of these compounds, found in fruits and vegetables, extend beyond aesthetic appeal: significant health benefits are observed due to their provitamin A activity [23]. Provitamin A carotenoids, including β-carotene, α-carotene, and β-cryptoxanthin, are converted into retinol, the biologically active form of vitamin A, in the human body [24]. Vitamin A is essential for a variety of physiological functions, including vision [25], immune function [26], cell growth and differentiation [27], and reproductive health [28].

In addition to provitamin A activity, carotenoids exhibit several other health benefits. They are effective antioxidants, scavenging free radicals that cause cellular damage and contribute to chronic diseases [29]. These compounds also demonstrate anti-inflammatory properties, which may help reduce inflammation in various health conditions [30]. Current research suggests that carotenoids may play a role in the prevention of cardiovascular disease, type 2 diabetes, bone diseases, and skin and eye diseases [31]. The consumption of these bioactive substances from plants often occurs in unprocessed forms, showcasing natural consumption or utilization for nectars/juices and preservation purposes [32]. To optimize food production, minimize by-product generation, and enhance profits, utilization of vegetable leaves to develop high-value ingredients and products holds significant potential [33]. 

The Azores Archipelago, with its unique environmental factors, is pivotal in leafy vegetable production in Portugal [34]. Characterizing bioactive compounds in agro-food plants and by-products from the Azores region in particular is essential to recognize their economic value in different industries. 

This work aims to characterize the whole leaves of seven different naturalized plants from the Azores region, namely *Annona cherimola* Mill., *Ipomoea batatas* (L.) Lam., *Colocasia esculenta* (L.) Schott, *Eriobotrya japonica* (Thunb.) Lindl., *Cymbopogon citratus* (DC.) Stapf, *Psidium guajava* L., and *Smallanthus sonchifolius* (Poepp.) H.Rob. The rationale behind the selection of these particular species stems from their widespread consumption and traditional use in folk medicinal practices, which implies a historical record of safe usage. Notably, recent studies have demonstrated the absence of toxicity in tea infusions derived from *A. Cherimola* leaves [35] and have even highlighted its potential to reduce diabetes [36], diarrhea, worm treatment and respiratory disorders in traditional medicine [37]. Similarly, *I. batatas* and *C. esculenta* leaves, also used for tea production [38] and widely used in the Azores region as a side dish for meals, have undergone toxicity testing, confirming their safety for human consumption [39]. Additionally, *E. japonica* and *C. citratus*, known for their traditional medicinal uses [40,41], have been shown to be non-toxic in animal studies [42,43,44]. *P. guajava* [45] and *S. sonchifolius* [46] have a long history of traditional medicine use and are also currently used in food supplements and tea infusions. This characterization is performed by analyzing chlorophyll, carotenoid, total phenol, flavonoid, and *ortho*-diphenols content, as well as antioxidant capacity using DPPH, ABTS, and FRAP methods. Polyphenol identification and quantification are assessed using HPLC–DAD in line with Directive (EU) 2018/851, contributing to the European transition to a circular economy.

## 2. Materials and Methods

### 2.1. Chemicals and Reagents

Potassium hydroxide, Folin–Ciocalteu’s reagent, gallic acid (3,4,5-trihydroxybenzoic acid) and acetic acid (both extra pure (>99%)), and sodium hydroxide (98%) were purchased from Panreac (Panreac Química S.L.U., Barcelona, Spain). Sodium nitrite, aluminum chloride, and sodium carbonate (all extra pure (>99%)) and ethanol were purchased from Merck (Merck, Darmstadt, Germany). Sodium molybdate (99.5%) was purchased from Chem-Lab (Chem-Lab N.V., Zedelgem, Belgium). Catechin (98%), Trolox (6-hydroxy-2,5,7,8-tetra-methylchroman-2-carboxylic acid, ≥98.0%), DPPH^•^ (2,2-diphenyl-1-picrylhidrazyl radical, ≤100.0%), ABTS^•+^ (2,2-azino-bis (3-ethylbenzothiazoline-6-sulphonic acid) diammonium salt ≥98.0%), potassium persulfate (K_2_S_2_O_8_, ≥99.0%), TPTZ (2,4,6-Tripyridyl-s-Triazine, ≥98.0%), and iron (III) chloride (FeCl_3_) (≥99.9%) were obtained from Sigma-Aldrich (Steinheim, Germany). Distilled water (Millipore, Bedford, MA, USA) was used for all extractions and analyses. Formic acid was obtained from Panreac (Castellar del Vallés, Barcelona, Spain). Acetonitrile was provided by J.T. Baker (Philipsburg, NJ, USA).

### 2.2. Sampling

The leaves of *A. cherimola*, *I. batatas*, *C. esculenta*, *E. japonica*, *C. citratus*, *P. guajava*, and *S. sonchifolius* plants were harvested in the Azores Archipelago, specifically on São Miguel Island, throughout the year 2021. Subsequently, the leaves were transported to the laboratory, frozen at −80 °C and lyophilized. The leaves were finely ground into a powder, standardized using 60 Tyler mesh sieves, and then stored under refrigeration conditions (6 ± 2 °C), shielded from light, until the time of analysis.

Each sample was analyzed in triplicate (*n* = 3) for each protocol.

### 2.3. Chlorophyll and Carotenoid Content

For the pigment quantification, 5 mg of each sample was incubated with 80% acetone for 24 h at 4 °C, followed by centrifugation at 5000 rotations per minute (rpm) for 10 min at 4 °C. Chlorophyll a, chlorophyll b, and carotenoids were quantified at 663.2 nm, 646.6 nm, and 470 nm, respectively, using the classical spectrophotometric method with a spectrophotometer (Thermo Electron Corporation, UVG 141604, Horsham and Loughborough, England). The pigment content of chlorophyll a, chlorophyll b, total chlorophyll (a + b), and carotenoids was calculated based on the method described by Lichtenthaler et al. [47] using Equations (1)–(4). The pigment content was expressed in μg/mL of the pigment extract solution. The results were then expressed in μg/g DW, considering the initial weight of the leaves. Data were reported as the average value of three replicates ± standard deviation.

Chlorophyll a (Ca) = 12.25 × A_663_ − 2.55 × A_646_ [μg/mL] (1)

Chlorophyll b (Cb) = 20.31 × A_646_ − 4.91 × A_663_ [μg/mL] (2)

Total chlorophyll (a + b) = 17.76 × A_646_ + 7.34 × A_663_ [μg/mL] (3)

Carotenoids = (1000 × A_470_ − 1.82 × Ca − 85.02 × Cb)/198 [μg/mL] (4)

### 2.4. Phenolic Extract Preparation

To prepare the phenolic extracts, 40 mg of samples were mixed with 1.5 mL of ethanol (EtOH)/distilled water (dH_2_O) (70:30, *v*/*v*). The mixture was vortexed and subsequently subjected to agitation at room temperature (RT) for 30 min. Following this, the mixture underwent centrifugation at 4 °C for 15 min at 1500 rpm to separate the supernatants from the solid residue. This extraction process was repeated three times, and the supernatants from each extraction round were collected and stored in a 5 mL volumetric flask at 4 °C. These extracts were utilized for determining the phenolic content and antioxidant capacity.

### 2.5. Determination of the Phenolic Content

The phenolic content from the seven leaf extracts was assessed using spectrophotometric methodologies outlined previously [48], with slight adaptations. A 96-well microplate format (PrimeSurface MS-9096MZ, Frilabo, Maia, Portugal) was employed, and absorbances were recorded using microplate readers (Multiskan GO Microplate Photometer, Thermo Fisher Scientific, Vantaa, Finland). 

#### 2.5.1. Total Phenolic Content

The total phenolic content of the seven extracts was determined using the Folin–Ciocalteu colorimetric method as previously described [49]. In brief, 20 µL of gallic acid standard solutions ranging from 0 mg/L to 250 mg/L or samples were individually added to each well of the microplate individually and mixed with 100 μL of the Folin–Ciocalteu reagent (1:10 dH_2_O). Then, 80 μL of 7.5% sodium carbonate (Na_2_CO_3_) was added. The reaction was incubated in an oven at 40–45 °C for 30 min in the dark. Absorbance was measured at 750 nm. Results were expressed in mg of gallic acid per gram of DW (mg GA/g DW) using gallic acid as a standard (5–200 mg/L). Data were reported as the average value of three replicates ± standard deviation.

#### 2.5.2. *Ortho*-Diphenols Content

The *ortho*-diphenols content of the seven extracts was determined by adding 160 μL of gallic acid standard solutions ranging from 0 mg/L to 250 mg/L or samples appropriately diluted to 40 μL of 50 g/L sodium molybdate (Na_2_MoO_4_) prepared in 50% EtOH. Mixtures were vortexed and incubated for 15 min at RT in the dark. The absorbance was measured at 375 nm and quantified using gallic acid as a standard (5–200 mg/L). Results were expressed in mg GA/g DW. Data were reported as the average value of three replicates ± standard deviation. 

#### 2.5.3. Flavonoid Content

The flavonoid content of the seven extracts was determined by adding 24 μL of catechin standard solutions ranging from 0 mg/L to 250 mg/L or samples appropriately diluted to 28 μL of 50 g/L sodium nitrite (NaNO_2_). After a 5 min incubation period at RT, 28 μL of 100 g/L aluminium chloride (AlCl_3_) was added, and the reaction was incubated for 6 min. Following this incubation period, 120 μL of 1.0 M sodium hydroxide (NaOH) was added. After a 30 s shaking, the absorbance was immediately read at 510 nm in the microplate reader and quantified using catechin as a standard (5–200 mg/L) and the results were expressed in mg of catechin per gram of DW (mg CAT/g DW). Data were reported as the average value of three replicates ± standard deviation.

### 2.6. Determination of the Antioxidant Capacity

The free radical scavenging capacity was assessed using the ferric-reducing antioxidant power (FRAP), ABTS and DPPH spectrophotometric methods following the procedures outlined by Queiroz et al. [13] and Gouvinhas et al. [50], with some modifications. 

#### 2.6.1. FRAP Assay

The FRAP assay was determined according to the methodology described by Yu et al. [51]. In total, 20 µL of the extracts was mixed with 180 µL of FRAP working solution (a mixture of TPTZ (10 mM dissolved in hydrochloric acid), ferric chloride (20 mM in water), and acetate buffer (300 mM, pH 3.6) in a ratio of 1:1:10). The reaction was incubated at 37 °C for 30 min in the dark. Following this period, the absorbance was read at 593 nm, and Trolox was employed as a standard in a range from 0.039 mmol/L to 1.250 mmol/L. The results were expressed in mmol Trolox equivalent per g of dry weight (mmol T/g DW) and data were reported as the average value of three replicates ± standard deviation. 

#### 2.6.2. DPPH Assay

For the DPPH method, a working solution of 8.87 mM DPPH^•^ radical in EtOH/dH_2_O (70:30, *v*/*v*) was prepared until reaching an absorbance of 1.00 ± 0.02 at 520 nm. The radical scavenging activity was determined by mixing 10 µL of the extract and 190 µL of the DPPH working solution for 30 min at RT in the dark. After this period, the absorbance at 520 nm was measured, with 70% hydroethanol (*v*/*v*) used as the blank. The DPPH radical scavenging activity assay was carried out as in the work of Domínguez-Perles et al. [12] with some modifications. To 10 μL of the sample or Trolox standard (from 0.039 mmol/L to 1.250 mmol/L), 190 μL of the DPPH solution was added.

The mixture was placed in the dark at room temperature for 30 min, and absorbance was measured at 520 nm in a microplate reader. Inhibition of free radical DPPH in percentages (%) was calculated using the following formula:

% inhibition = 100 × (Abs_520_ blank − Abs_520_ sample)/Abs_520_ blank (5)

DPPH radical scavenging activity of the samples was determined by interpolation of the calibration curve for Trolox. Results were expressed in (mmol T/g DW).

#### 2.6.3. ABTS^•+^ Assay

ABTS^•+^ radicals were generated by reacting 5 mL of 7.0 mM ABTS stock solution in water with 88 µL of 140 mM potassium persulfate (K_2_S_2_O_8_) in water. After a 16 h incubation in the dark, the ABTS solution was diluted to a working solution with 100 mM sodium acetate buffer (pH 4.5) to achieve a final absorbance of 0.70 ± 0.02 at 734 nm. A Trolox calibration curve was prepared by diluting a 5 mM Trolox stock standard in EtOH/dH_2_O (1:1 *v*/*v*) until reaching 0.11 mM. Trolox was employed as a standard in a range from 0.034 mmol/L to 0.200 mmol/L. Then, 2 mL of the ABTS working solution was added and the final volume of 200 μL was filled using dH_2_O. The radical scavenging capacity was determined by adding 100 μL of each hydroethanolic extract, 100 μL of dH_2_O, and 2 mL of the ABTS working solution. The inhibition percentage was calculated for each standard solution and sample using the provided Equation (5). dH_2_O served as the blank, and the absorbance of the mixture was measured at 734 nm to determine the radical scavenging capacity. Results were expressed in mmol Trolox/g DW and data were reported as the average value of three replicates ± standard deviation.

### 2.7. Determination and Quantification of Phenolic Compounds by HPLC–DAD

The polyphenolic composition and quantification of leaf samples were evaluated using HPLC–DAD, following a previously described methodology [49]. Briefly, the HPLC mobile phase consisted of two solvents: solvent A, which was composed of water containing 0.1% trifluoroacetic acid (TFA) (99.9:0.1, *v*/*v*), and solvent B, which was composed of acetonitrile with 0.1% TFA (99.9:0.1, *v*/*v*). A linear gradient elution scheme was employed as follows: starting with 0% solvent B at 0 min, maintaining 0% solvent B until 5 min, increasing to 20% solvent B at 15 min, further increasing to 50% solvent B at 30 min, reaching 100% solvent B at 45 min, maintaining 100% solvent B until 50 min, returning to 0% solvent B until 55 min, and finally maintaining 0% solvent B until 60 min. Between each sample, solvent B concentration was returned to 0% from 55 min to 60 min to stabilize the system and prepare the column for subsequent analysis. Each extract sample was subjected to analysis at a temperature of 25 °C using a C18 column (250 × 46 mm, 5 μm, ACE HPLC Columns, Advanced Chromatography Technologies Ltd., Abeerden, Scotland, UK). The analysis was performed with a flow rate of 1.0 mL/min, and a sample injection volume of 20 μL was utilized. Chromatograms were recorded for benzoic acids and flavan-3-ols at wavelengths of 254 nm and 280 nm, respectively. For cinnamic acids, the recording was conducted at 320 nm, while for flavonoids, it was carried out at 370 nm. Peak retention time, UV spectra, UV max absorbance bands, and comparison with external commercial standards (Extrasynthese, Cedex, Genay, France) were used to identify the individual polyphenols in the extracts. For the quantification of each polyphenol, the internal standard method was employed. Consequently, external standards were prepared in a solution of EtOH/dH_2_O (70:30, *v*/*v*) at a concentration of 1.0 mg/mL. These standards were run in parallel with the samples using HPLC–DAD. All samples were injected in triplicate, and the concentrations of phenolic compounds were expressed in mg/mL.

### 2.8. Statistical Analysis

Statistical comparisons were analyzed in SPSS Statistics, version 27.0.1.0 (IBM SPSS Statistics Software, Chicago, IL, USA), and GraphPad Prism, version 8.4.0 (GraphPad Software, San Diego, CA, USA). Normality was measured by the Shapiro–Wilk statistical test and was assumed when *p* > 0.05. When normality was achieved, analysis of variance (ANOVA), followed by a post hoc Tukey test, was applied to detect differences between the content of chlorophylls, carotenoids, total phenolics, ortho-diphenols, flavonoids, FRAP, ABTS, DPPH, and quantification of phenolic compounds by HPLC-DAD. Principal component analysis (PCA) was performed using the mean values of the triplicates in the JMP Statistical Discovery^TM^, version 11.0.0 (Neil Hodgson). The data were adjusted to a range of 0–100 while accounting for the highest mean value determined during each experiment. Outliers were identified and excluded, taking into consideration the ROUT method (Q = 10%). The results were presented as mean ± standard deviation (SD). All experiments were carried out in triplicate and the level of statistical significance was considered at *p* < 0.05.

Heat mapping of Pearson’s correlations (commonly used to express the strength between two continuous variables, which is useful for demonstrating mathematical relation of the response variables and to understand the proportion of the fluctuation of one variable that was predictable from the other variable) with respective statistical significances was performed in the software OriginPro 2022 v.9.9.0.225 to understand the nature and degree of inter-relationship among the individual phenolic compounds identified and the antioxidant capacity.

## 3. Results

### 3.1. Quantitative Analysis of Chlorophylls and Carotenoids

**Figure 1:**
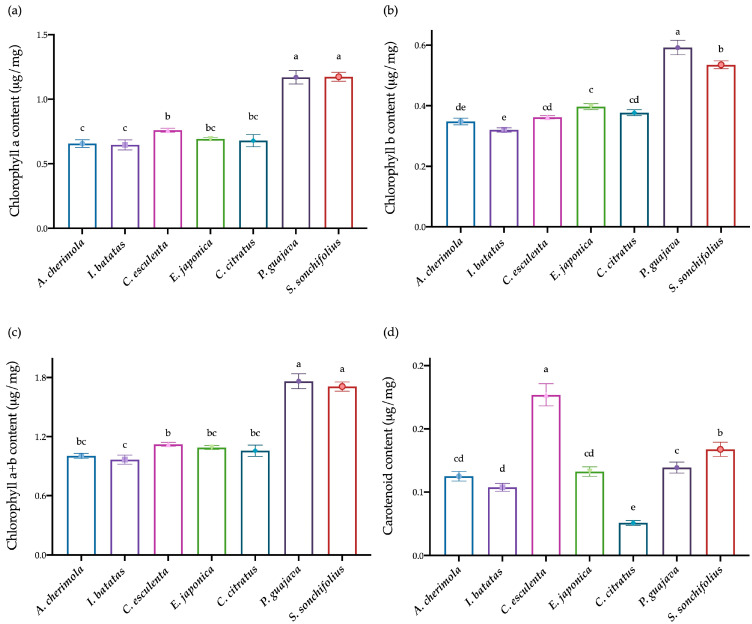
Chlorophyll and carotenoid content of leaf extracts from the Azores region. (**a**) Chlorophyll a content (μg/mg), (**b**) chlorophyll b content (μg/mg), (**c**) total chlorophyll (a + b) content (μg/mg), and (**d**) carotenoid content (μg/mg) measured in *A. cherimola*, *I. batatas*, *C. esculenta*, *E. japonica*, *C. citratus*, *P. guajava*, and *S. sonchifolius*. (*n* = 3 per leaf). Data are present as mean ± SD. Different letters correspond to significant differences between varieties (*p* < 0.05). ANOVA followed by a post hoc Tukey test.

The antioxidant and anti-inflammatory properties of chlorophylls and carotenoids have recently gained traction in the cosmetic industry, where they are utilized to enhance UV radiation protection and address inflammatory-related skin conditions [52,53]. Recognizing this industrial trend and considering the lack of characterization of leaves from the Azores region, we first conducted an evaluation of the content of chlorophylls and carotenoids in seven leaf extracts from this region (Figure 1). The aim was to uncover their potential for reuse and to enhance the economic value of these plant by-products in different industrial sectors. 

We observed variations in the content of chlorophyll a, with significantly higher levels found in *P. guajava* (L.) (1.17 ± 0.05 μg/mg), and *S. sonchifolius* (1.17 ± 0.04 μg/mg) extracts. Subsequently, *C. esculenta* (L.) Schott (0.76 ± 0.02 μg/mg) exhibited a higher content than *E. japonica* (0.69 ± 0.01 μg/mg) and *C. citratus* (0.68 ± 0.05 μg/mg), with no significant differences observed between them. *A. cherimola* (0.66 ± 0.03 μg/mg) and *I. batatas* (0.65 ± 0.04 μg/mg) presented the lowest content of chlorophyll a, and no significant differences were found compared to *E. japonica* and *C. citratus* (Figure 1a). 

Interestingly, this pattern was consistently observed for chlorophyll b and total chlorophylls as well (Figure 1b,c). Once again, *P. guajava* (chlorophyll b − 0.59 ± 0.02 μg/mg; chlorophyll a + b − 1.76 ± 0.08 μg/mg) and *S. sonchifolius* (chlorophyll b − 0.54 ± 0.01 μg/mg; chlorophyll a + b − 1.71 ± 0.05 μg/mg) exhibited higher values. However, contrary to the total chlorophyll content, the levels of chlorophyll b between *P. guajava* and *S. sonchifolius* were significantly different. Following this, *E. japonica* (0.40 ± 0.01 μg/mg) displayed higher chlorophyll b content, but it was not significantly different from *C. esculenta* (L.) Schott (0.36 ± 0.01 μg/mg) and *C. citratus* (0.38 ± 0.01 μg/mg). These two leaf extracts showed no significant differences with *A. cherimola* (0.35 ± 0.01 μg/mg). *I. batatas* (0.32 ± 0.01 μg/mg) presented the lowest value of chlorophyll b, but not significantly different from *A. cherimola* (Figure 1b). 

Once again, *P. guajava* (1.76 ± 0.08 μg/mg) and *S. sonchifolius* (1.71 ± 0.05 μg/mg) presented higher contents of total chlorophylls, followed by *C. esculenta* (L.) Schott (1.12 ± 0.02 μg/mg), *E. japonica* (1.09 ± 0.02 μg/mg), *C. citratus* (1.06 ± 0.06 μg/mg), *A. cherimola* (1.00 ± 0.03 μg/mg), and *I. batatas* (0.97 ± 0.05 μg/mg). Significant differences were observed only between *P. guajava* and *S. sonchifolius*, as well as between *C. esculenta* and *I. batatas* (Figure 1c). 

On the other hand, the content of carotenoids was significantly higher in *C. esculenta* (0.20 ± 0.01 μg/mg), followed by *S. sonchifolius* (0.13 ± 0.01 μg/mg), *P. guajava* (0.11 ± 0.01 μg/mg), *E. japonica* (0.11 ± 0.01 μg/mg), *A. cherimola* (0.10 ± 0.01 μg/mg), *I. batatas* (0.09 ± 0.01 μg/mg), and *C. citratus* (0.04 ± 0.00 μg/mg). The carotenoid content of *E. japonica* and *A. cherimola* was not significantly different between *P. guajava* and *I. batatas* (Figure 1c). On the other hand, significant differences were observed between these last two species.

Overall, our data conclusively revealed that *P. guajava* and *S. sonchifolius* displayed elevated chlorophyl content, whereas *C. esculenta* demonstrated heightened levels of carotenoids.

### 3.2. Phenolic Content and Antioxidant Capacity

In response to the growing recognition of the potential health benefits associated with plant-derived phenolic compounds, we proceeded to the quantification of the phenolic content and antioxidant capacity of leaves from the plant species native to Azores described above.

As depicted in Table 1, in terms of total phenols, *A. cherimola* exhibited the highest content and was significantly different from the remaining leaf extracts (176.54 ± 11.50 mg GA/g dry weight (DW)), *ortho*-diphenols (232.84 ± 1.85 mg GA/g DW), and flavonoids (79.02 ± 2.18 mg CAT/g DW). *E. japonica* (111.58 ± 6.08 mg GA/g DW) and *P. guajava* (145.85 ± 1.42 mg GA/g DW) showed the second-highest content of total phenols and *ortho*-diphenols, respectively, with no significant differences in flavonoid content between these two species. 

The total phenol content of *C. citratus* (27.90 ± 2.22 mg GA/g DW) was not significantly different from that of *C. esculenta* (26.99 ± 2.27 mg GA/g DW) and *I. batatas* (20.06 ± 1.23 mg GA/g DW), and the latter two species did not significantly differ from *S. sonchifolius* (11.18 ± 0.88 mg GA/g DW).

*E. japonica* (137.00 ± 1.11 mg GA/g DW) exhibited the third highest content of *ortho*-diphenols. However, the levels between *C. esculenta* (80.10 ± 1.32 mg GA/g DW), *C. citratus* (79.01 ± 1.08 mg GA/g DW), and *I. batatas* (78.56 ± 1.76 mg GA/g DW) were not significantly different. *S. sonchifolius* (46.44 ± 1.52 mg GA/g DW) showed a significantly lower content of *ortho*-diphenols.

The flavonoid content of *C. citratus* (12.01 ± 0.85 mg CAT/g DW) was not significantly different from that of *I. batatas* (11.24 ± 1.16 mg CAT/g DW). Moreover, the latter was not significantly different from *C. esculenta* (6.03 ± 0.33 mg CAT/g DW), and the former was not significantly different from *S. sonchifolius* (3.80 ± 0.24 mg CAT/g DW) (Table 1).

Subsequently, we assessed the antioxidant capacity of the seven leaf extracts from Azores using three distinct methodologies to ascertain any potential correlation with the phenolic content. As depicted in Table 2, *A. cherimola* displayed a significantly higher antioxidant capacity regarding FRAP (1.02 ± 0.02 mmol Trolox/g), DPPH (0.43 ± 0.03 mmol Trolox/g), and ABTS (0.35 ± 0.01 mmol Trolox/g) assays. *P. guajava* exhibited the second better results in FRAP (0.47 ± 0.01 mmol Trolox/g) and DPPH (0.27 ± 0.01 mmol Trolox/g) assays, although in this last assay, the antioxidant capacity of *P. guajava* did not significantly differ from that of *E. japonica*. The same was not observed between these two species in the ABTS assay, where *E. japonica* exhibited a significantly higher antioxidant capacity (0.22 ± 0.01 mmol Trolox/g) than *P. guajava* (0.18 ± 0.01 mmol Trolox/g). 

These outcomes aligned with the phenolic content results, underscoring that *A. cherimola*, *P. guajava*, and *E. japonica* presented the highest values.

In the case of the other plant species, noteworthy differences were observed. The FRAP values of *C. citratus* (0.12 ± 0.00 mmol Trolox/g) were significantly different from those of *I. batatas* (0.07 ± 0.00 mmol Trolox/g), *C. esculenta* (0.05 ± 0.00 mmol Trolox/g), and *S. sonchifolius* (0.04 ± 0.00 mmol Trolox/g). 

However, concerning this parameter, *I. batatas* (0.07 ± 0.00 mmol Trolox/g) did not show significantly different results from those exhibited by *C. esculenta* (0.05 ± 0.00 mmol Trolox/g), which, in turn, did not present significantly different results from those of *S. sonchifolius* (0.04 ± 0.00 mmol Trolox/g).

Conversely, no significant differences were detected in the DPPH assay between *C. citratus* (0.08 ± 0.00 mmol Trolox/g), *I. batatas* (0.05 ± 0.00 mmol Trolox/g), and *C. esculenta* (0.05 ± 0.00 mmol Trolox/g), and the latter two were not significantly different from *S. sonchifolius* (0.04 ± 0.00 mmol Trolox/g). However, *C. citratus* (0.05 ± 0.00 mmol Trolox/g) exhibited significant differences compared to *I. batata* (0.04 ± 0.00 mmol Trolox/g), *C. esculenta* (0.04 ± 0.00 mmol Trolox/g), and *S. sonchifolius* (0.02 ± 0.00 mmol Trolox/g) relative to ABTS results (Table 2).

**Figure 2:**
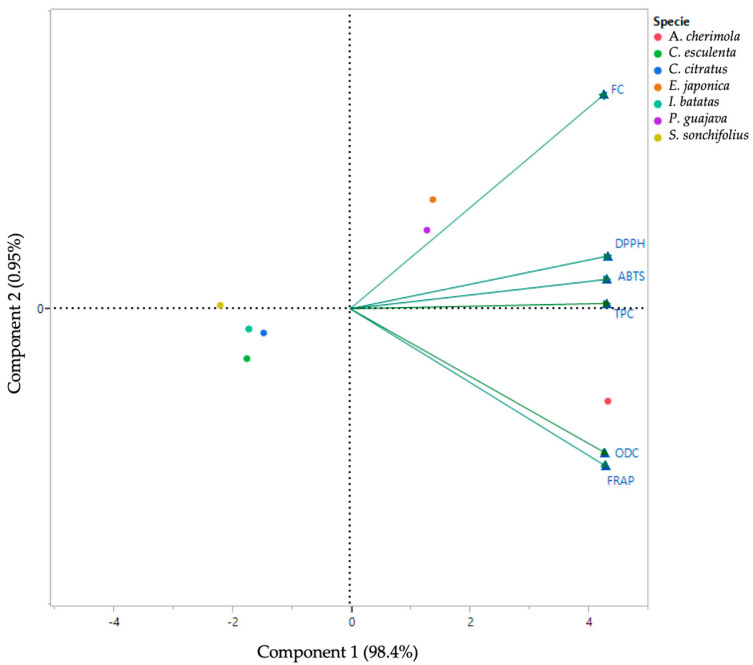
Principal component analysis (PCA) scores and loading plots of total phenol content (TPC), *ortho*-diphenols content (ODC), flavonoid content (FC), and antioxidant capacity (FRAP, DPPH, and ABTS) of *A. cherimola* (red), *I. batatas* (light blue), *C. esculenta* (green), *E. japonica*, *C. citratus* (dark blue), *P. guajava* (purple), and *S. sonchifolius* (yellow). ABTS: Scavenging capacity of the ABTS radical, DPPH: Scavenging capacity of the DPPH radical, FC: Flavonoid content, FRAP: Ferric-reducing antioxidant power, ODC: *Ortho*-diphenols content, TPC: Total phenolic content.

Principal Component Analysis (PCA) was employed to examine the clustering patterns of the plants under study regarding phenolic content and antioxidant capacity. As illustrated in Figure 2, the scatter plot of PCA applied to the results concerning the spectrophotometric assays reveals intriguing insights. Notably, the first two-dimensional components, PCA1 and PCA2, accounted for 98.40% and 0.95% of the loading score, respectively. In the right quadrant, *A. cherimola*, *P. guajava*, and *E. japonica* stood out with the highest values. *A. cherimola* was particularly distinguished in the lower right quadrant, exhibiting elevated values across all analyses, including total phenol, *ortho*-diphenols, and flavonoid content, as well as antioxidant capacity using FRAP, DPPH, and ABTS. The proximity between ODC and FRAP in the lower right quadrant corroborates the results previously shown in Table 1 and Table 2, which enables us to closely correlate these two parameters.

Conversely, in the lower left quadrants were *C. esculenta*, *C. citratus*, *I. batatas*, and *S. sonchifolius*. The plants in this set were placed close to each other and in opposite sides of the scores plot in relation to the different phenolic composition parameters and the antioxidant capacity assays, findings that once again are in line with the previously observed results shown in Table 1 and Table 2. 

### 3.3. Identification and Quantification of Phenolic Compounds by HPLC–DAD

**Figure 3:**
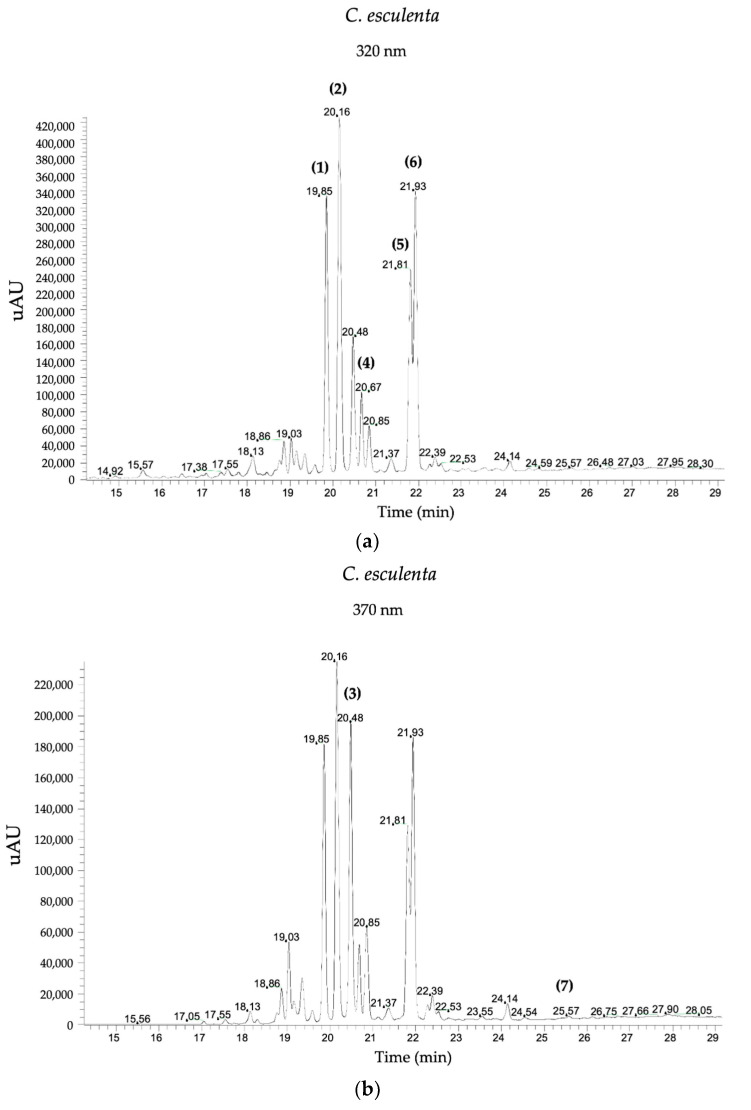
Representative HPLC–DAD chromatogram of *C. esculenta* Azorean leaf extracts, corresponding to two distinct wavelengths: (**a**) 320 nanometers (nm) and (**b**) 370 nm. Peak (1) corresponds to Apigenin, (2) to Apigenin derivative isomer 1, (3) to Quercetin-3-*O*-glucoside, (4) to Apigenin derivative isomer 2, (5) to Apigenin derivative isomer 3, (6) to Apigenin derivative isomer 4, and (7) to Luteolin. (*n* = 3 per leaf).

The characterization of phenolic compounds in leaf extracts was carried out using HPLC–DAD methodology. Detailed information on retention time and concentration of identified phenolic compounds in mg/100 g DW is presented in Table 3. Our study successfully identified and quantified a total of twenty phenolic compounds in the extracts, encompassing diverse classes such as hydroxycinnamic acids, flavan-3-ols, flavonols, and flavones. A representative HPLC–DAD chromatogram of C. esculenta is presented in Figure 3. Noteworthy is the distinctiveness in profiles observed among leaf extracts from the seven Azorean plants, with statistically significant variations noted between the plant species. Concerning hydroxycinnamic acids, the results illustrated in Table 3 revealed the absence of any compounds belonging to this class in *C. esculenta* leaves. In stark contrast, the leaf extract of *E. japonica* stood out with notably elevated levels of neochlorogenic acid (15.33 ± 0.34 mg/100 g DW) and chlorogenic acid (22.21 ± 0.60 mg/100 g DW), distinguishing itself significantly from the remaining. In the realm of flavonoids, specifically within the category of flavonols, the compound catechin was exclusively identified and quantified in *I. batatas* (4.79 ± 0.08 mg/100 g DW). Also regarding flavonols, *A. cherimola* drew attention by exhibiting significantly elevated levels of all four identified compounds in this class (Quercetin-3-*O*-rutinoside, Quercetin-3-*O*-glucoside, Quercetin-3-*O*-rhamnoside, and Isorhamnetin). The levels observed in these species proved to be markedly distinct from all others, reinforcing the notion of a high flavonoid content, as previously determined by colorimetric methods for this leaf. Once more, the extract of *C. esculenta* (L.) Schott distinguished itself, revealing the exclusive presence and quantification of Quercetin-3-*O*-glucoside at a concentration of 3.70 ± 0.05 mg/100 g DW, with no detection of other flavonols identified in the remaining plants.

Regarding the flavones, a subclass of flavonoids boasting the highest count of identified and quantified compounds (eight), the extract of *C. esculenta* takes center stage in stark contrast. Notably, this leaf unveiled the identification and quantification of six out of the eight compounds, and from these six, five are Apigenin and Apigenin Derivatives.

### 3.4. Pearson Correlation Analysis

**Figure 4:**
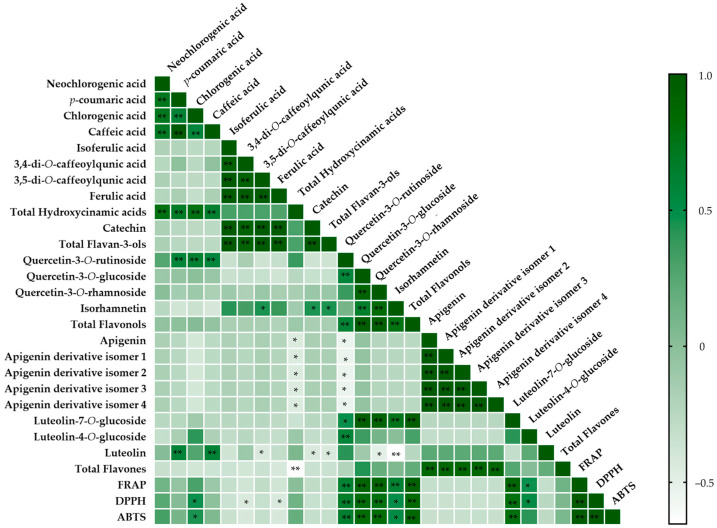
Heatmap of correlations between individual phenolic compounds and antioxidant capacity as shown in Supplementary Table S1. Statistically significant correlations: * *p* < 0.05, ** *p* < 0.01.

To elucidate the intricate relationship between the phenolic composition and the antioxidant capacity of the different leaf extracts, we conducted a comprehensive analysis employing Pearson’s correlation coefficient [54,55] (Figure 4 and Supplementary Materials Table S1). Upon scrutinizing Figure 4 and Table S1, a discernible pattern emerges, revealing strong positive correlations (*p* < 0.01) among the three distinct methodologies utilized for evaluating antioxidant capacity, namely between FRAP and ABTS (r = 0.979), FRAP and DPPH (r = 0.978), and ABTS and DPPH (r = 0.987).

The overarching findings underscore that the antioxidant properties of the different leaf extracts, as evaluated by the three different methodologies, exhibit significant strong positive correlations (*p* < 0.01) with total flavonols (FRAP r = 0.918; DPPH r = 0.840; ABTS r = 0.863). This consistent trend extends to all the individual compounds within this class, particularly with quercetin-3-*O*-glucoside, quercetin-3-*O*-rhamnoside, and quercetin-3-O-rutinoside as evidenced by strong positive correlations with all three methodologies, as depicted in Table S1.

Additionally, it is noteworthy that within the flavone class, significant positive correlations (*p* < 0.01) were uncovered between luteolin-7-*O*-glucoside and antioxidant capacity, specifically r = 0.889, r = 0.792, and r = 0.789 with FRAP, DPPH, and ABTS, respectively. Still, in the flavone class, significant positive correlations were observed, albeit with lower r values, namely between luteolin-4-*O*-glucoside, FRAP (r = 0.468), and DPPH (r = 0.526).

## 4. Discussion

Chlorophylls and carotenoids, essential to the photosynthetic process, play a crucial role in human health, offering potential applications across diverse biological contexts and in the development of value-added foods [16,56]. Their characterization in Azorean plants presents a compelling avenue for research, given the unique characteristics of the Azorean archipelago. However, there is a dearth of literature evidence regarding the evaluation of chlorophyll and carotenoid content in Azores plants, including *A. cherimola*, *I. batatas*, *C. esculenta*, *E. japonica*, *C. citratus*, *P. guajava*, and *S. sonchifolius*. This study pioneers the assessment of pigment content in these leaf plant extracts. Particularly noteworthy are the elevated levels of chlorophylls in *P. guajava* and *S. sonchifolius*, while *C. esculenta* exhibited higher carotenoid content. Beyond chlorophylls and carotenoids, phenolic compounds have been associated with various health benefits due to their potent biological properties, encompassing anti-inflammatory, antiallergic, anticancer effects, and positive impacts on cardiovascular function [57]. In this study, we extended our evaluation to include the identification and quantification of phenolic compounds and antioxidant capacity in the aforementioned leaf plant extracts. To the best of our knowledge, no specific studies have been conducted to assess the content of chlorophylls and carotenoids in *A. cherimola*, *E. japonica*, and *S. sonchifolius* leaf extracts. While one study reported carotenoid levels in *A. cherimola* pulp extracts collected in Italy, direct comparison is limited as it is primarily focused on fruit pulp rather than leaves [58]. Similarly, we identified a study on chlorophyll content, but the experimental design precluded a direct comparison with our results [59]. Additionally, no studies employing the same methodology were found for the content of chlorophylls and carotenoids in *I. batatas* [60,61,62] and *C. citratus* leaves [63]. There are few studies on the quantification of carotenoids and chlorophylls in *P. guajava* leaves. However, studies have been conducted on pulp and fruit, making them unsuitable for comparison in the present study [64,65].

Notably, our study revealed elevated values of phenolic content in *A. cherimola* compared to other research. For instance, Mannino et al. [35] reported an average total phenol content of 6.00 ± 1.32 mg GA/g DW. Remarkably, our results are, on average, 28% higher (176.54 ± 11.50 mg GA/g DW). Zengin et al. [66] achieved a higher total phenol content in *I. batatas* leaf extracts from Northern Italy using three distinct extraction methods, namely soxlet (77.39 ± 0.99 mg GA/g DW), microwave (73.10 ± 3.75 mg GA/g DW), and decoction (89.26 ± 1.34 mg GA/g DW), so we cannot make a direct comparation. Pawlowska et al. [67] reported a lower total phenol content (0.48 ± 0.11 mg GA/g DW) in *E. japonica* leaves, collected in Pisa, Italy, than in our study (111.58 ± 6.08 mg GA/g DW). Hong et al. [68] conducted a study on phenolic content in different species of *E. japonica* collected in China, also reporting total phenolic content values (47.5 ± 1.7–54.9 ± 2.4 mg GA/g DW) lower than those in our study (111.58 ± 6.08 mg GA/g DW). Studies on *C. citratus* leaves collected in Punjab, Pakistan report total phenols (1.32 ± 1.12 mg GA/g DW) lower than our results (27.90 ± 2.22 mg GA/g DW) [69]. In Díaz-de-Cerio et al.’s study [70] on *P. guajava* leaves from Motril (Spain) at three oxidation stages (high, medium, and low, respectively), total phenol values (87.91 ± 0.05 mg GA/g DW, 92.0 ± 0.4 mg GA/g DW, and 103 ± 2 mg GA/g DW, respectively) were similar to our study (88.65 ± 7.76 mg GA/g DW), with closer approximation in the highest oxidation stage. The studies by Camarena-Tello et al. [71] and Maryam et al. [72] in *P. guajava* leaves involved the use of different experimental procedures, making them impossible to compare with our results. Russo et al. [73] studied the phenolic profile of *S. sonchifolius* leaves collected from different soils, revealing a total phenol content ranging from 58.63 ± 1.04 mg GA/g DW to 91.07 ± 1.41 mg GA/g DW. These values are higher than the ones observed in our study (11.18 ± 0.88 mg GA/g DW). However, it is essential to note that the authors, despite utilizing a methodology similar to ours, measured absorbance at 723 nm, whereas we measured it at 750 nm. This difference in measured wavelengths could account for the disparity in results. No studies to date have investigated the total phenol content in *C. esculenta* leaves.

Regarding the *ortho*-diphenols content, we have not found studies in the literature regarding the quantification of this parameter in all the plant leaves in this study. Furthermore, none of these studies have used the methodologies employed in our research to evaluate the flavonoid content in the leaves of *A. cherimola*, *E. japonica*, and *P. guajava*. Zengin et al. [66] evaluated the flavonoid content in *I. batatas*, but a direct comparison is not feasible due to the utilization of rutin as the standard. Rustiana et al. [74] quantified the flavonoid content in the ethanolic extract of *C. esculenta* leaves from Indonesia, reporting a value of 4.33 ± 0.03 mg quercetin/g. Nonetheless, a direct comparison of this result is challenging due to the utilization of quercetin as the standard. A study on *C. citratus* leaves collected in Punjab, Pakistan showed flavonoid content (0.91 ± 0.81 mg CAT/g DW) lower than our results (12.01 ± 0.85 mg CAT/g DW, respectively) [69]. A study also assessed flavonoid content in *S. sonchifolius* leaves, but the methodologies employed differed from those in our study [73]. Ueda et al. [75] obtained elevated values of flavonoids (7.06–11.40 mg CAT/g DW) for *S. sonchifolius* leaves compared to our results (3.80 ± 0.24 mg CAT/g DW). However, the authors increased the temperature to 100 °C during leaf extraction, which could cause degradation of phenolic compounds and chemical alteration modifying their natural composition [76].

No studies to date have investigated the antioxidant capacity in *A. cherimola*, *C. citratus*, and *P. guajava* leaves. The unique study using FRAP, DPPH, and ABTS methodologies for measuring the antioxidant capacity of *I. batatas* leaves used different extraction methodologies, preventing a direct comparison of the results [66]. A study conducted by Gonçalves et al. [77] determined the antioxidant capacity using the DPPH assay in different *C. esculenta* varieties under different soil irrigation conditions from São Miguel Island, Azores. However, it is not possible to compare these results with ours as they are expressed in IC50 values. Singh et al. [63] assessed the antioxidant capacity using the DPPH method in *C. esculenta* from North, Middle, and South Andaman and Nicobar during the late rainy season. However, a direct comparison of the results is not possible as they were determined using IC50 values. Studies reported ABTS levels (0.74 ± 3.78 mmol Trolox/g DW) higher than ours (0.22 ± 0.01 mmol Trolox/g DW) in *E. japonica* leaves collected in Pisa, Italy [67]. Conversely, Hong et al. [68] obtained FRAP values (0.4 ± 19.5–0.5 ± 17.7 mmol Trolox/g DW) within the range of our values (0.43 ± 0.01 mmol Trolox/g DW). The methodologies employed in *S. sonchifolius* leaves to assess the antioxidant capacity were different from those in our study [46,78,79].

In the investigation conducted by Mannino et al. [58], seven cultivars of *A. cherimola* were examined. In contrast to our findings, these researchers did not detect any compounds classified as phenolic acids. However, they did identify catechin, a result not replicated in our study. Regarding quercetin-3-*O*-glucoside and quercetin-3-*O*-rutinoside (both falling within the flavonol class), the concentrations ranged from 791.53 mg/100 g DW to 2388.35 mg/DW and 1015.96 mg/100 DW to 3167.09 mg/100 g DW, respectively. Irrespective of the specific cultivar under investigation, the concentrations reported by Mannino et al. [58] consistently exceeded those observed in our study for these particular compounds. In the investigation conducted by Díaz-de-Cerio et al. [80], chlorogenic acid was identified in the leaves of the studied species, a finding consistent with our own observations. Additionally, quercetin-3-*O*-rutinoside was similarly identified by these authors. However, it is noteworthy that Díaz-de-Cerio et al. [80] solely identified these compounds without quantifying them individually, preventing us from making a direct comparison of concentrations. Furthermore, it is crucial to acknowledge the disparity in the analytical technique employed, with HPLC-ESI-TOF-MS utilized in their study. Consequently, discrepancies in both the quantity and profile of identified compounds are anticipated due to methodological divergence. It is noteworthy to emphasize that the flavonol class exhibited the highest overall expression in the leaves of these species in our study. However, luteolin-7-*O*-glucoside predominated as the quantitatively most abundant compound in the leaves of *A. cherimola*. Notably, this compound, along with luteolin-4-*O*-glucoside, constituted the exclusive representatives of the flavone class identified in our investigation.

In the case of *I. batatas* leaves, Fu et al. [81] explored various solvents and extraction ratios, including EtOH/H_2_O (70:30 *v*/*v*) (the same as utilized in our study). They observed a pronounced presence of hydroxycinnamic acids, akin to our findings. However, it is noteworthy that the quantities reported by these authors for these compounds were higher than those observed in our study. Specifically, they reported concentrations of 303 mg/100 g DW, 173 mg/100 g DW, and 917 mg/100 g DW for 3-caffeoylquinic acid, 3,4-dicaffeoylquinic acid, and 3,5-dicaffeoylquinic acid, respectively. Nevertheless, these authors identified only one flavonol, namely quercetin-3-*O*-hexosylhexoside (a compound that remained unidentified in our study). In contrast, our investigation identified four quercetin derivatives, notably highlighting isorhamnetin (3′-*O*-methyl-quercetin). Furthermore, our study unveiled the presence of catechin, the exclusive representative of the flavan-3-ols class, uniquely identified in *I. batatas* leaves. Additionally, no flavones were discerned in the leaves of this species in our study.

Concerning the characterization of the remaining leaves in this study, it is noteworthy that there is scant information in the literature, and there are limited studies on the species *P. guajava*, *E. japonica*, *S. sonchifolius*, and *C. citratus*.

In the case of *E. japonica* leaves, Pawlowska et al. [67] identified and quantified twenty-five compounds belonging to the classes of phenolic acids, flavones, and flavonols. The content is expressed in mg/100 g DW of sample, with the most significant differences found in the total phenolic acids. In our study, these were quantified in higher amounts compared to the findings reported by Pawlowska et al. [67] (42.48 ± 0.26 mg/100 g DW and 21.34 ± 0.34 mg/100 g DW, respectively) despite the identification of fewer compounds (chlorogenic acid, neochlorogenic acid, *p*-coumaric acid, and caffeic acid). 

Concerning *C. citratus* leaves, Costa et al. [82] identified and quantified twelve phenolic compounds using HPLC. Notably, in comparison with the present study, their research revealed a higher content of flavonoids. However, in our investigation, only three flavones, namely luteolin, luteolin-7-*O*-glucoside, and luteolin-4-*O*-glucoside, were identified and quantified at concentrations of 4.11 ± 0.16 mg/100 g DW, 4.09 ± 0.05 mg/100 g DW, and 2.36 ± 0.25 mg/100 g DW, respectively.

The disparity in the phenolic profile between these two studies is likely associated with the fact that these compounds are secondary metabolites that are produced by the plant under stress conditions [83]. Additionally, in our study, the plants are still in the adaptability phase to the climate and soil conditions of the Azores region. The leaves analyzed were obtained from younger trees, fully justifying the observed differences.

The same holds true for the limited studies described in the literature regarding the phenolic composition of *C. esculenta*. Noteworthy in the recent study by Shehata et al. [84] in which eleven phenolic compounds were identified and quantified, predominantly phenolic acids, a compound class not identified by us. The most abundant compound quantified in their study was *p*-coumaric acid, with a concentration of 46.49 mg/100 g DW of the sample.

In contrast to the study described, in our work, the class of compounds identified in the greatest number and quantity was the flavone class (apigenin, derivative isomer 1, 2, 3 and 4 from apigenin, and luteolin, in the total amount of 55.20 ± 0.32 mg/100 g DW).

Regarding the other two leaf species under study, *P. guajava* and *S. sonchifolius*, the literature data are even more scarce, with (to the best of our knowledge) only one study described for each species. For the species *P. guajava*, Diaz-de-Cerio et al. [85] employed HPLC-DAD-ESI-QTOF-MS analyses in the negative mode to identify seventy-three phenolic compounds.

Surprisingly, none of these seventy-three compounds were detected by us. However, we identified and quantified phenolic acids (17.97 ± 0.76 and 2.87 ± 0.22 mg/100 g DW), flavonols (7.50 ± 0.14 and 0.77 ± 0.01 mg/100 g DW), and flavones (13.55 ± 0.48 and 1.91 ± 0.01 mg/100 g DW), which were not identified by Diaz-de-Cerio et al. [85].

The same trend profile is observed in the case of the *S. sonchifolius* species, where the only study found in the literature, as described by Russo et al. [73], only resembles the present study in the identification and quantification of chlorogenic acid and caffeic acid.

The incorporation of Heat Map analysis, employing Pearson correlations, stands as an important methodological approach in comprehending the dynamic interrelation between the antioxidant capacity of leaf extracts and the quantified phenolic compounds within the different species. This analytical tool provides a nuanced understanding of the specific chemical contributors driving antioxidant capacity.

The findings derived from the current investigation concerning Pearson correlations, as depicted in Figure 4 and elaborated in Table S1, substantiate the elevated antioxidant efficacy inherent in compounds categorized under the flavonol class. Moreover, these results align with the information articulated in Table 2 and Table 3, concerning the antioxidant potential and phenolic composition of the different leaf extracts. Specifically, the leaf extracts of *A. Cherimola*, which markedly surpassed others in all of the conducted antioxidant capacity assessments, concurrently demonstrated total flavonol levels at least threefold higher than those observed in the remaining extracts. Moreover, the extract from this particular species demonstrated notably elevated levels of luteolin-7-*O*-glucoside, approximately 4.8 times higher than the subsequent species in the order of magnitude, namely *P. guajava*. This compound (luteolin-7-*O*-glucoside), in turn, displayed robust and meaningful correlations with antioxidant capacity, as assessed through three distinct methodologies.

Flavonols, identified as potent antioxidants, possess the ability to safeguard cells against oxidative stress, thereby reducing the likelihood of developing chronic conditions such as cancer, heart disease, and diabetes [86]. These compounds exhibit high antioxidant capacity due to specific structural features. Several studies have highlighted the significance of the 4-keto function, the 2,3 double bond, and specific hydroxyl groups, such as the 3-OH group, in enhancing the antioxidant capacity of flavonols [87,88,89,90,91,92]. Numerous research studies have explored the antioxidant potential of flavonols and flavones derived from leaf extracts of various botanical sources. For instance, in a study conducted by Cao et al. [93], *Cyclocarya paliurus* (Batal.) Iljinskaja leaf extracts yielded results akin to the present study. The researchers observed significant positive correlations between total quercetin glycosides and DPPH (r = 0.73), FRAP (r = 0.86), and ABTS (r = 0.91).

In a study by Cezarotto et al. [94], a strong positive correlation was found between the content of quercetin-3-*O*-rutinoside and the DPPH assay while assessing the antioxidant properties of *Vaccinium ashei* leaf extracts.

Contrastingly, Orak et al. [95], scrutinizing *Olea europaea* L. leaf extracts, identified significant positive correlations between FRAP and luteolin-7-*O*-glucoside (*r* = 0.728) and luteolin-4-*O*-glucoside (*r* = 0.833). In contrast to our study, these authors discovered a negative correlation between DPPH and these two flavones, specifically *r* = −0.570 and *r* = −0.544 with luteolin-7-*O*-glucoside and luteolin-4-*O*-glucoside, respectively.

Undoubtedly, this study on the potential adaptation of certain tropical plants to the climate of the Azores region is of paramount importance. Given the limited existing research on the leaves of these species, and considering both their phenolic content and distinctive profiles, these plants could hold significant potential within the realms of circular economy and industrial symbiosis. This could elevate their value, particularly in the cosmetic and pharmaceutical industries. 

## 5. Conclusions

Overall, our comprehensive investigation into the pigment composition, antioxidant properties, and polyphenolic profile of leaf extracts from different Azorean plant leaves shed light on their potential as rich sources of bioactive compounds. This study demonstrated that *P. guajava* and *S. sonchifolius* possess elevated chlorophyll content, while *C. esculenta* exhibited higher carotenoid levels. Notably, *A. cherimola*, *E. japonica*, and *P. guajava* displayed increased content of total phenols, *ortho*-diphenols, and flavonoids, aligning with enhanced antioxidant capacities determined through FRAP, DPPH, and ABTS assays. High-performance liquid chromatography with diode array detection characterization revealed distinct profiles, with *E. japonica* and *C. citratus* extracts exhibiting higher levels of hydroxycinnamic acids. In sharp contrast, *A. cherimola* presented a higher content of flavonoids, corroborating the results obtained in colorimetric assays. Our preliminary study aimed to delve into the bioactive compounds present in these plant species and explore their potential health-promoting properties. Taken together, this work highlighted the potential of some Azores plant leaves as a novel source of antioxidants, offering prospects for further natural product development for different industries including cosmetic, food, and nutraceutical. Further scientific investigation to validate their safety and nutritional properties, particularly in the context of functional food development, is needed. 

## Figures and Tables

**Table 1:** Total phenolic, *ortho*-diphenols and flavonoid content of *A. cherimola*, *I. batatas*, *C. esculenta*, *E. japonica*, *C. citratus*, *P. guajava*, and *S. sonchifolius* Azorean leaf extracts.

Plant Species	TPC (mg GA/g DW)	ODC (mg GA/g DW)	FC (mg CAT/g DW)
*A. cherimola*	176.54 ± 11.50 ^a^	232.84 ± 1.85 ^a^	79.02 ± 2.18 ^a^
*I. batatas*	20.06 ± 1.23 ^de^	78.56 ± 1.76 ^d^	11.24 ± 1.16 ^cd^
*C. esculenta*	26.99 ± 2.37 ^de^	80.10 ± 1.32 ^d^	6.03 ± 0.33 ^de^
*E. japonica*	111.58 ± 6.08 ^b^	137.00 ± 1.11 ^c^	54.99 ± 4.84 ^b^
*C. citratus*	27.90 ± 2.22 ^d^	79.01 ± 1.08 ^d^	12.01 ± 0.85 ^c^
*P. guajava*	88.65 ± 7.76 ^c^	145.85 ± 1.42 ^b^	56.46 ± 0.39 ^b^
*S. sonchifolius*	11.18 ± 0.88 ^e^	46.44 ± 1.52 ^e^	3.80 ± 0.24 ^e^

CAT: Catechin, DW: Dry weight, FC: Flavonoid content, GA: Gallic acid, ODC: *Ortho*-diphenols content, TPC: Total phenol content (*n* = 3 per leaf). Data are present as mean ± SD. Different letters in the same column correspond to significant differences between species (*p* < 0.05). ANOVA followed by a post hoc Tukey test.

**Table 2:** Antioxidant capacity of *A. cherimola*, *I. batatas*, *C. esculenta*, *E. japonica*, *C. citratus*, *P. guajava*, and *S. sonchifolius* Azorean leaf extracts using FRAP, DPPH, and ABTS methods.

Plant Species	FRAP (mmol T/g)	DPPH (mmol T/g)	ABTS (mmol T/g)
*A. cherimola*	1.02 ± 0.02 ^a^	0.43 ± 0.03 ^a^	0.35 ± 0.01 ^a^
*I. batatas*	0.07 ± 0.00 ^e^	0.05 ± 0.00 ^cd^	0.04 ± 0.00 ^e^
*C. esculenta*	0.05 ± 0.00 ^ef^	0.05 ± 0.00 ^cd^	0.04 ± 0.00 ^ef^
*E. japonica*	0.43 ± 0.01 ^c^	0.25 ± 0.00 ^b^	0.22 ± 0.01 ^b^
*C. citratus*	0.12 ± 0.00 ^d^	0.08 ± 0.00 ^c^	0.05 ± 0.00 ^d^
*P. guajava*	0.47 ± 0.01 ^b^	0.27 ± 0.01 ^b^	0.18 ± 0.01 ^c^
*S. sonchifolius*	0.04 ± 0.00 ^f^	0.04 ± 0.00 ^d^	0.02 ± 0.00 ^f^

ABTS: Scavenging capacity of the ABTS radical, DPPH: Scavenging capacity of the DPPH radical, FRAP: Ferric-reducing antioxidant power, T: Trolox. (*n* = 3 per leaf). Data are present as mean ± SD. Different letters in the same column correspond to significant differences between species (*p* < 0.05). ANOVA followed by a post hoc Tukey test.

**Table 3:** Identification and quantification of phenolic compounds present in leaf plant extracts from the Azores Region by HPLC-DAD.

Rt	λ (nm)	Identified Compounds	Quantification (mg/100 g DW)						
			*A. cherimola*	*I. batatas*	*C. esculenta*	*E.* *japonica*	*C.* *citratus*	*P.* *guajava*	*S.* *sonchifolius*
**Hydroxycinnamic acids**									
16.60	320	Neochlorogenic acid	ND	ND	ND	15.33 ± 0.34 ^a^	ND	ND	0.64 ± 0.01 ^b^
18.09	320	*p*-Coumaric acid	ND	ND	ND	2.18 ± 0.03 ^a^	1.79 ± 0.18 ^b^	ND	ND
18.35	320	Chlorogenic acid	3.96 ± 0.17 ^d^	2.63 ± 0.05 ^e^	ND	22.21 ± 0.60 ^a^	7.79 ± 0.26 ^c^	17.97 ± 0.76 ^b^	1.36 ± 0.02 ^f^
18.64	320	Caffeic acid	ND	ND	ND	2.76 ± 0.05 ^a^	2.44 ± 0.06 ^b^	ND	0.26 ± 0.01 ^c^
19.92	320	Isoferulic acid	ND	10.06 ± 0.19 ^a^	ND	ND	0.73 ± 0.03 ^b^	ND	ND
25.62	320	3,4-di-*O*-caffeoylqunic acid	ND	4.70 ± 0.25 ^a^	ND	ND	1.99 ± 0.01 ^b^	ND	ND
27.25	320	3,5-di-*O*-caffeoylqunic acid	ND	2.08 ± 0.09 ^a^	ND	ND	ND	ND	ND
28.38	320	Ferulic acid	ND	6.56 ± 0.06 ^a^	ND	ND	1.37 ± 0.07 ^b^	ND	0.61 ± 0.03 ^c^
		**Total**	3.96 ± 0.17	26.03 ± 0.13	ND	42.48 ± 0.26	16.11 ± 0.10	17.97 ± 0.76	2.87 ± 0.20
**Flavan-3-ols**									
19.05	280	Catechin	ND	4.79 ± 0.08 ^a^	ND	ND	ND	ND	ND
		**Total**	ND	4.79 ± 0.08	ND	ND	ND	ND	ND
**Flavonols**									
20.19	370	Quercetin-3-*O*-rutinoside	4.25 ± 0.15 ^b^	0.80 ± 0.01 ^d^	ND	3.87 ± 0.09 ^c^	4.99 ± 0.08 ^a^	3.82 ± 0.10 ^c^	0.33 ± 0.02 ^e^
20.46	370	Quercetin-3-*O*-glucoside	12.34 ± 0.18 ^a^	1.16 ± 0.04 ^d^	3.70 ± 0.05 ^c^	3.49 ± 0.10 ^c^	4.30 ± 0.37 ^b^	3.68 ± 0.18 ^c^	0.28 ± 0.00 ^e^
20.99	370	Quercetin-3-*O*-rhamnoside	8.23 ± 0.07 ^a^	0.98 ± 0.01 ^c^	ND	1.69 ± 0.02 ^b^	ND	ND	0.16 ± 0.01 ^d^
22.40	370	Isorhamnetin	4.70 ± 0.22 ^a^	3.04 ± 0.20 ^b^	ND	ND	ND	ND	ND
		**Total**	29.52 ± 0.16	5.98 ± 0.07	3.70 ± 0.05	9.05 ± 0.07	9.29 ± 0.23	7.50 ± 0.14	0.77 ± 0.01
**Flavones**									
19.85	320	Apigenin	ND	ND	12.57 ± 0.31 ^a^	ND	ND	ND	ND
20.16	320	Apigenin derivative isomer 1	ND	ND	17.18 ± 0.22 ^a^	ND	ND	ND	ND
20.67	320	Apigenin derivative isomer 2	ND	ND	2.78 ± 0.13 ^a^	ND	ND	ND	ND
21.81	320	Apigenin derivative isomer 3	ND	ND	8.92 ± 0.87 ^a^	ND	ND	ND	ND
21.93	320	Apigenin derivative isomer 4	ND	ND	11.53 ± 0.31 ^a^	ND	ND	ND	ND
22.34	370	Luteolin-7-*O*-glucoside	25.92 ± 1.52 ^a^	ND	ND	ND	4.09 ± 0.05 ^b^	5.38 ± 0.30 ^b^	1.21 ± 0.01 ^c^
23.66	370	Luteolin-4-*O*-glucoside	2.78 ± 0.40 ^b^	ND	ND	ND	2.36 ± 0.25 ^b^	6.26 ± 1.13 ^a^	0.28 ± 0.01 ^c^
25.28	370	Luteolin	ND	ND	2.22 ± 0.06 ^b^	1.60 ± 0.07 ^d^	4.11 ± 0.16 ^a^	1.91 ± 0.00 ^c^	0.42 ± 0.01 ^e^
		**Total**	28.70 ± 0.96	ND	55.20 ± 0.32	1.60 ± 0.07	10.56 ± 0.15	13.55 ± 0.48	1.91 ± 0.01

ND: Not detected, Rt: Retention time. In the same row, different letters correspond to significant differences between leaf plant extracts (*p* < 0.05), according to ANOVA followed by a post hoc Tukey test. Data are present as mean ± SD (*n* = 3 per leaf).

## Data Availability

Data are contained within the article and Supplementary Materials.

## Supplementary Materials

The following supporting information can be downloaded at: https://www.mdpi.com/article/10.3390/antiox13030325/s1, Table S1: Correlation matrix–Pearson’s correlation factor values (r).
